# Growth Hormone Mediates Its Protective Effect in Hepatic Apoptosis through Hnf6

**DOI:** 10.1371/journal.pone.0167085

**Published:** 2016-12-09

**Authors:** Kewei Wang, Minhua Wang, Maureen Gannon, AiXuan Holterman

**Affiliations:** 1 Department of Surgery/Pediatric Surgery, U of Illinois College of Medicine, Peoria, IL, United States of America; 2 Departments of Medicine/Molecular Physiology/Biophysics, Vanderbilt University Medical Center, Nashville, TN, United States of America; National Institutes of Health, UNITED STATES

## Abstract

**Background and Aims:**

Growth hormone (GH) not only supports hepatic metabolism but also protects against hepatocyte cell death. *Hnf6* (or *Oc1)* belonging to the Onecut family of hepatocyte transcription factors known to regulate differentiated hepatic function, is a GH-responsive gene. We evaluate if GH mediates Hnf6 activity to attenuate hepatic apoptotic injury.

**Methods:**

We used an animal model of hepatic apoptosis by bile duct ligation (BDL) with *Hnf6* -/- (KO) mice in which hepatic *Hnf6* was conditionally inactivated. GH was administered to adult wild type WT and KO mice for the 7 days of BDL to enhance Hnf6 expression. *In vitro*, primary hepatocytes derived from KO and WT liver were treated with LPS and hepatocyte apoptosis was assessed with and without GH treatment.

**Results:**

In WT mice, GH treatment enhanced Hnf6 expression during BDL, inhibited Caspase -3, -8 and -9 responses and diminished hepatic apoptotic and fibrotic injury. GH-mediated upregulation of Hnf6 expression and parallel suppression of apoptosis and fibrosis in WT BDL liver were abrogated in KO mice. LPS activated apoptosis and suppressed Hnf6 expression in primary hepatocytes. GH/LPS co-treatment enhanced Hnf6 expression with corresponding attenuation of apoptosis in WT-derived hepatocytes, but not in KO hepatocytes. ChiP-on-ChiP and electromobility shift assays of KO and WT liver nuclear extracts identified *Ciap1* (or *Birc2*) as an Hnf6-bound target gene. Ciap1 expression patterns closely follow Hnf6 expression in the liver and in hepatocytes.

**Conclusion:**

GH broad protective actions on hepatocytes during liver injury are effected through Hnf6, with Hnf6 transcriptional activation of *Ciap1* as an underlying molecular mediator.

## Introduction

Growth Hormone (GH) regulates somatic growth, cell regeneration and metabolic activities. GH actions are mediated by Stat5 transcriptional activation of *Igf-1* [1] and its downstream mediators, with the liver as a major site of Igf-1 production. GH-Stat5 pathway is also implicated in regulating the promoter function of Hepatocyte nuclear factor *Hnf6* (Onecut; Oc-1) [2], a liver-enriched transcription factor critical to normal liver development, differentiation and function. Null mice with global disruption of Hnf6 have aberrant hepatoblast maturation, abnormal bile duct formation and die early of cholestasis [3]. In the differentiated liver, Hnf6 regulates hepatocyte target genes involved in metabolic [4] [5] [6] and regenerative functions [7] [8]. We previously treated mice with recombinant human GH to increase *in vivo* Hnf6 expression and found that GH treatment improved bile duct ligation (BDL) hepatic injury, enhanced cholesterol clearance and improved hepatocyte-specific cellular proliferation [8]. GH has also previously been shown to attenuate hepatocyte death. In GH receptor *Ghr*-deficient mice, cholic acid feeding worsens cholestasis and hepatocyte apoptosis [9]. Additionally, compared to *mdr2* (multi-drug resistant transporter-2) null mutant mice, cholestasis, hepatic fibrosis and hepatocyte apoptosis were exacerbated in *mdr2*/*Ghr* or *mdr2*/*Stat5* double null mice [9] [10] [11]. In these GH-resistant mice, Hnf6 expression was diminished, suggesting that increased susceptibility to hepatic apoptosis in the absence of GH function can be attributed to impaired Hnf6 hepatocyte-specific function, and that Hnf6 biological function is broader than the previously demonstrated Hnf6 regulation of hepatocyte proliferation and metabolic activities. We therefore test the hypothesis that GH apoptosis function during cholestatic liver injury is critically linked to Hnf6 function in hepatocytes. We used mice where *Hnf6* was conditionally inactivated in the liver (*Hnf6* -/-, referred to as KO mice) to evaluate if Hnf6 deficiency 1) worsens hepatic apoptotic injury; 2) impairs GH pro-survival effects in an *in vivo* model of hepatocyte apoptosis by BDL cholestatic injury; and 3) suppresses Hnf6 hepatic target genes underlying GH-mediated function.

## Methods

### Materials

Human recombinant GH was obtained from the NIDDK National Hormone and Peptide Program. Mouse monoclonal Hnf6; rabbit polyclonal antibodies against mouse procaspase-3, -8, -9 and cleaved caspases -3, -8, -9, α-Sma, and Igf1 were from Santa Cruz Biotechnology, CA; mouse anti-b-actin, rabbit anti-phospho-Stat5 A/B antibodies were from Sigma-Aldrich; Ciap1, pStat5, rabbit anti-mouse horseradish peroxidase, and goat anti-mouse horseradish peroxidase were from Cell Signaling Technology.

### Hnf6 conditional null mice *Hnf6* -/- (KO)

Mice homozygous for LoxP-containing *Hnf6* allele (*Hnf6*^flox/flox^) [12] were interbred with transgenic mice expressing Cre-recombinase under the control of the *albumin* enhancer/promoter to generate *Hnf6*^flox/flox^; *albumin*-Cre animals. A*lbumin*-Cre allowed Cre-mediated liver-specific recombination and inactivation of both floxed *Hnf6* alleles to generate the KO mice.

### Animal procedures

The animal study protocol was approved by and conducted in accordance with the Institutional Animal Care and Use Committee (IACUC) at the University of Illinois College of Medicine. Six to eight weeks F6 generation mice received care according to the IACUC guidelines. Following bile duct ligation (BDL n = 8–10, Sham n = 4), PBS or human recombinant GH was delivered at 5 ug/h by subcutaneous Alzet miniosmotic pumps for 1 week [8]. Because male mice were used in our previous work, as well as in *Ghr* and *Stat5* null models, and because of the higher sensitivity of male mice to GH-responsive hepatic target genes [13,14], male mice were used.

### Immunostaining

Paraffin-embedded liver tissues underwent TUNEL staining using TdT-FragEL^TM^ DNA fragmentation kit from Calbiochem (#QIA33), or α-Sma immunostaining [15]. The strength of α-Sma staining was quantified by Image J analysis program. The percentage of TUNEL-positive hepatocytes in 30 random microscopic fields for 1000 hepatocytes/mouse was counted.

### Western blot assays

In three independent experiments, liver total or nuclear protein extract immune complexes [15] were detected with horseradish-conjugated secondary antibody (Fisher) followed by chemiluminescence (ECL + plus, Amersham Biosciences, Inc.).

### Chromatin Immunoprecipitation (ChIP) Assays

Briefly [7], liver (n = 3/group) was homogenized, fixed in 1% Formaldehyde; and fragmented DNA samples from sonicated crude nuclear extracts were immunoprecipitated without antiserum, with rabbit or Hnf6 antiserum. *Ciap1* forward and reverse primers were 5’-GAGCCTGGTGGTAGTGTGGT-3’ (-619/-599) and 5’-CCATGAGTGGGCTGATTTCT-3’ (-81/-61), *Xiap* forward and reverse primers were 5’-CCCAGATCCACCCACCTAAC-3’ and 5’-AACGAGCCTCAACCTCAGTC-3’ respectively.

### ChiP on ChIP

Hnf6-immunoprecipitated DNA samples (with non-specific IgG as control) from three wild type and three KO liver were hybridized to Affymetrix Mouse Promoter 1.0R array containing over 25,500 mouse promoter regions. Hnf6-bound genes were grouped into functional categories using the biological module–centric algorithm DAVID Bioinformatics Resources 6.7, provided by National Institute of Allergy and Infectious Diseases (NIAID), NIH (https://david.ncifcrf.gov/home.jsp). Genes with Enrichment Scores ≥ 1.25 (p ≤ 0.05) were further investigated by hierarchical cluster analysis using MAT (model-based analysis of tiling-array) scores. The data have been deposited at http://www.ncbi.nlm.nih.gov/geo/query/acc.cgi?acc=GSE80498 in MIAME-compliant standard format. (S1 Table and S1 Fig)

### Electrophoretic mobility shift assay (EMSA)

Crude nuclear extracts from WT and KO liver or *in vitro*-expressed Hnf6 proteins [16] were prepared with labeled double-stranded oligonucleotides 1, 2 and 3 bearing Hnf6-binding sites (Table 1). Protein-DNA complexes were separated from unbound DNA probes using 6% native polyacrylamide gels containing 0.5x Tris borate/EDTA in three independent experiments.

**Table 1:** Primer DNA sequences of real time PCR genes.

Name	Forward 5'-3'	Reverse 5'-3'
*Ciap1*	TCAGGTGATGTGGAGCTCAG	GCATACATCCCTGCACACAC
*Ciap2*	TCCCTGTCATCTCACCATGA	TGTCTAGCATCAGGCCACAG
*Xiap*	TTGGAACATGGACATCCTCA	TGCCCCTTCTCATCCAATAG
*Survivin*	GAGTGAGTCCCAGCTTCCAG	TGATTCCCAGAGATCGTTCC
*Livin*	ACGGTCAAAAGGAAGGGACT	CAGGCTGGGTCTCTCTTCTG
*Naip*	GCCAGGTACCATGAAGAGGA	AATTCACATTTGGGGAACCA
*Apollon*	CTGCGGGGTTGTGATTTACT	GCAGAGAGCATCCAACACAA
*Bcl-2*	CACGCTGGGAGAACAGGGTA	GGATGTACTTCATCACTATCTCCCG
*Bad*	AGGATCGCTGTGTCCCTTTA	GCAGTCCAGAACAGGAGAGG
*Bcl-x*	CCTTCAGGCCTCTCTCTCCT	CCAGCAGCTCCTCACACATA
*Bak-1*	CCAACATTGCATGGTGCTAC	AGGAGTGTTGGGAACACAGG
*Caspase-3*	AGCTTGGAACGGTACGCTAA	CGTACCAGAGCGAGATGACA
*Caspase-8*	CCTAGACTGCAACCGAGAGG	GCAGGCTCAAGTCATCTTCC
*Caspase-9*	TGCCCTTGCCTCTGAGTAGT	AACAAAGAAACGCCCACAAC
*Ctgf*	CAAAGCAGCTGCAAATACCA	GGCCAAATGTGTCTTCCAGT
*Tgfb2r*	CGGAAATTCCCAGCTTCTGG	TTTGGTAGTGTTCAGCGAGC

Forward primer and reverse primer sequences shown with position upstream of the transcription start site.

### Real-Time PCR

Total liver RNAs were extracted using RNA-STAT-60 (Tel-Test "B" Inc. Friendswood, TX). cDNA were amplified and analyzed in triplicate using a MyiQ Single Color Real-Time PCR Detection System (Biorad, Hercules, CA). Primer sequences are listed in Table 1. *Hnf6*, *Hnf1b*, *Hnf4a*, *Oc2*, *Foxa2*, *C/epb* primer sequences have been reported previously [6,15,17]. Relative gene expression was calculated using the mathematical delta delta method by PE Applied Biosystems. Levels were normalized to *Cyclophilin* and reported relative to group with the lowest expression level.

### Primary hepatocyte culture

Primary hepatocytes from 3–5 male KO or WT liver were isolated after collagenase perfusion, purified by Percoll centrifugation, plated at 5.5 x 10^5^ cells/well and cultured in DMEM supplemented with 100 nM dexamethasone, 100 nM insulin, 100 U/ml penicillin G, 100 mg/ml streptomycin, and 10% bovine calf serum at 37°C in 5% CO_2_. After three hours, LPS (1 ug/mL) and/or GH (1 ug/mL) were added for 32 hours before cell harvest.

### Statistical Analysis

Data are expressed as mean ± S.D.. Intergroup differences were evaluated by ANOVA for repeated measures. A p value of ≤ 0.05 is considered to be significant. All statistical analyses were performed with the software SPSS.

## Results

### 1. GH diminishes BDL apoptosis in WT but not KO liver

**Fig 1:**
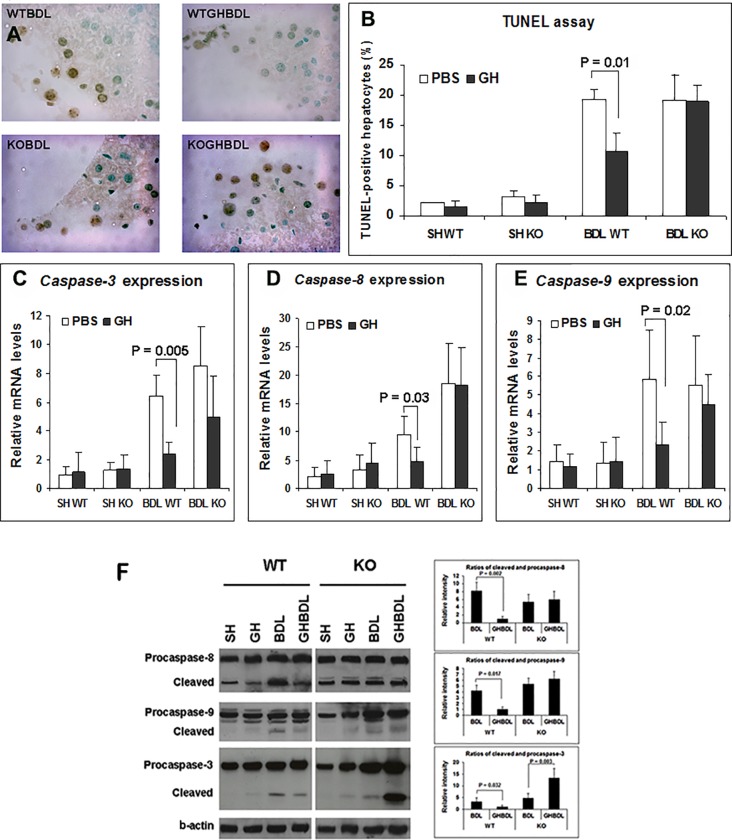
TUNEL assay and Caspase expression in WT and KO liver. (A) Representative TUNEL-labeled liver micrographs from Sham (SH) WT or KO mice without (WT, KO) or with GH treatment (WTGH and KOGH); and Bile duct ligation (BDL) WT or KO mice without (WTBDL, KOBDL) or with GH treatment (GHWTBDL and GHKOBDL) (n = 8–10/group). The arrows identify TUNEL + hepatocytes. (B) Bar graph of % TUNEL (+) hepatocytes/100x high power field from WT or KO SH or BDL liver treated with PBS (□) or GH (■) and significant p value. (C-E) *Caspase-3* (C), *-8* (D) and *-9* (E) in WT or KO liver treated with PBS (□) or GH (■) and significant p values. Gene levels were calculated relative to SH WT liver. (F) Representative western blots of Procaspase-8, -9, -3, cleaved proteins and b-Actin in SH or BDL WT and KO liver and the corresponding bar graphs of caspase staining intensity in BDL samples with significant p values.

**Fig 2:**
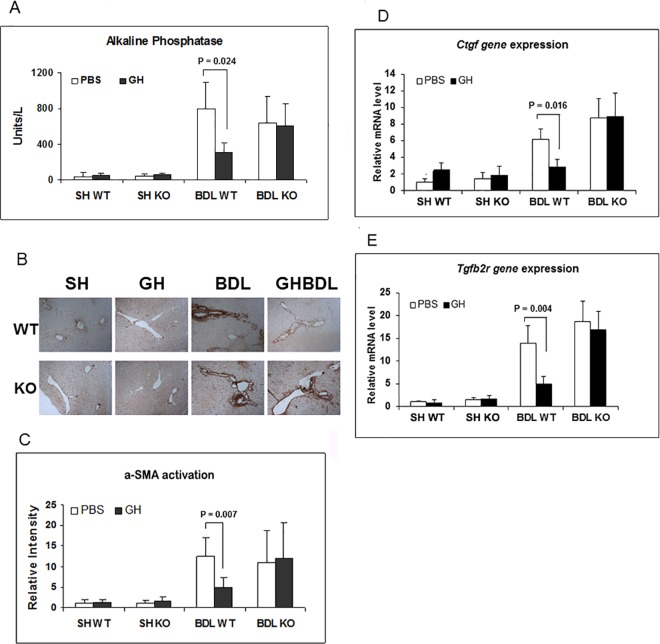
Hepatic cholestasis and fibrosis in WT and KO mice. (A) Serum Alkaline Phosphatase levels (a marker of cholestasis) in SH or BDL WT and KO mice treated with PBS (□) or GH (■) and significant p values. (B) Representative micrographs of α-Sma immunostaining of SH or BDL WT and KO liver. (C) Bar graph of α-Sma immunostaining of SH or BDL WT and KO liver treated with PBS (□) or GH (■) with intensity relative to SH WT and significant p value. (D) *Ctgf* expression in SH or BDL WT and KO mice treated with PBS (□) or GH (■) with levels relative to Sham WT and significant p values. (E) *Tgfb2R* expression in SH or BDL WT and KO mice treated with PBS (□) or GH (■) with levels relative to SH WT and significant p values.

Previously, we reported that GH infusion during BDL increases hepatic Hnf6 and Igf1 expression, with corresponding diminished cholestatic liver injury, enhanced hepatocyte proliferation and improved hepatic metabolic function [8]. Common features in hepatic cholestatic injury are hepatocyte cell death and hepatic fibrosis, with apoptosis driving the fibrogenesis response (19, 20). Experimental models of cholestasis in GH-resistant mice provide strong evidence for GH role in protecting against hepatocyte death [9,10,18–21]. To evaluate if Hnf6 directly regulates hepatocyte apoptosis and if Hnf6 mediates GH function in apoptosis, BDL was performed in *Hnf6* -/- (referred to as KO) mice with conditional inactivation of hepatic *Hnf6* in hepatocytes and biliary epithelial cells. As previously reported [22], at baseline, KO adult mice have normal liver biochemistries and no intrahepatic abnormality. Except for a 4-fold elevation (p = 0.02) in *Hnf1b*, a transcription factor regulated by Hnf6 [3], the expression of *Hnf6* paralogs *Oc-2* and *Oc-3* [23,24] and major hepatocyte nuclear factors such as *Hnf1a*, *Hnf4a*, *Foxa2*, and *C/ebp* are unaffected in KO mice (data not shown). Following 7 days of BDL injury, relative to Sham liver, PBS-treated BDL WT show enhanced hepatocyte apoptosis by TUNEL assay (Fig 1A and 1B), increased expression of *Caspase-3*, *-8* and *-9* by real-time PCR (Fig 1C and 1E), and increased cleaved caspase protein expression by western blot (Fig 1F). With GH treatment, TUNEL hepatocyte apoptosis is reduced (Fig 1A and 1B), as is Caspase-3, -8, -9 gene and protein expression in WT BDL liver (Fig 1C and 1F). This is paralleled by attenuated cholestasis with lower serum alkaline phosphatase [8] (Fig 2A), diminished α-smooth muscle actin (α-Sma) marker for fibrosis (Fig 2B and 2C) and profibrotic genes *Ctgf*, *Tgfb2R* (Fig 2D and 2E) in GH-treated BDL WT liver relative to PBS BDL liver. In the absence of GH, KO BDL and WT BDL hepatocytes show similar degree of apoptotic injury by TUNEL assay (Fig 1A and 1B); and in whole liver, similar real-time PCR of hepatic *Caspase-3*, *-8* and *-9* (Fig 1C–1E) and western blot for cleaved caspase protein expression (Fig 1F; with the exception of cleaved caspase-8). The extent of hepatic cholestasis (Fig 2A) and fibrosis (Fig 2B–2E) is also similar between WT BDL and KO BDL liver in the absence of GH. In KO mice, previously demonstrated GH-associated reduction of hepatocyte TUNEL labeling, and of hepatic *Caspase -3*, *-8*, *-9* expression in WT BDL liver is lost (Fig 1C–1E). In fact, we observed increased caspase-3 expression, minimal to no reduction of caspase-8, and -9 proteins (Fig 1F), undiminished cholestatic injury, no differences in *Ctfg*, *Tgfb2R* expression or α-Sma staining relative to PBS-treated KO BDL liver (Fig 2). The data suggest that Hnf6 is essential to GH-mediated reduction of BDL hepatocyte apoptosis, hepatic cholestasis and fibrosis.

### 2. GH reduction of BDL apoptosis is Hnf6-dependent

**Fig 3:**
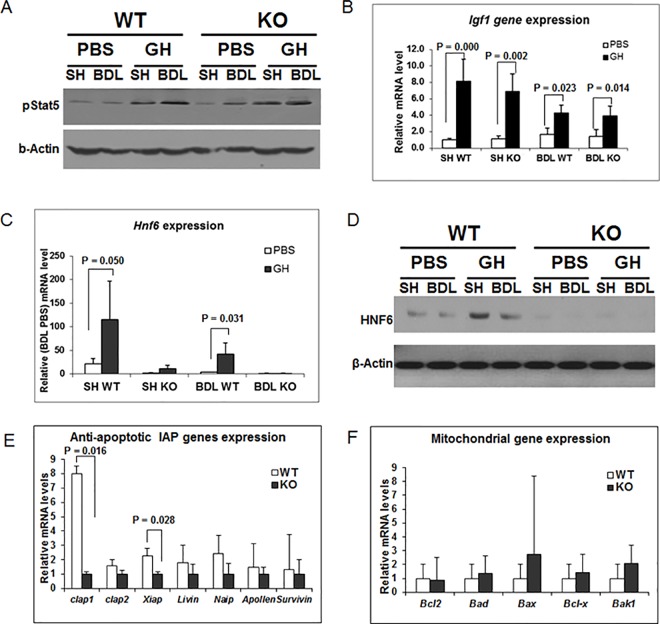
Downstream signaling response to GH treatment. (A) Representative western blot of phosphorylated Stat5 and b-Actin of liver nuclear proteins from Sham (SH) or BDL WT and KO liver treated with PBS or GH. (B) *Igf1* expression in WT SH and KO SH as well as WT BDL and KO BDL liver treated with PBS (□) or GH (■), with levels relative to SH KO PBS liver and significant p values. (C) *Hnf*6 expression in SH or BDL WT and KO liver treated with PBS (□) or GH (■), with levels relative to SH KO liver and significant p values. (D) Representative western blot of Hnf6 nuclear protein extracts from SH or BDL WT and KO liver treated with PBS or GH. (E) Baseline expression levels of Iap (*Ciap1*, *Ciap2*, *Xiap*, *Livin*, *Naip*, *Apollen and Survivin*) family of genes from WT and KO liver with levels shown relative to KO liver and significant p values. (F) Baseline expression levels of Bcl2 (*Bcl2*, *Bad*, *Bax*, *Bcl-x*, *Bak1*) families of genes from WT and KO liver with levels shown relative to KO liver and significant p values.

Since GH transcriptionally activates *Hnf6* and *Igf1*, we assessed their contribution to GH protective effects. Following GH infusion in WT mice, GH signaling is enhanced as shown by higher hepatic phosphorylated Stat5 protein in GH-Sham relative to PBS-Sham liver; and similarly, in GH-BDL relative to PSB-BDL liver (Fig 3A). Correspondingly, GH-responsive *Igf1* (Fig 3B), and *Hnf6* gene (Fig 3C) and protein expression (Fig 3D) increase in GH-treated Sham WT relative to PBS-treated WT Sham; and similarly, in GH-BDL relative to PSB-BDL liver. Previous publication indeed showed that GH-induced increase of Hnf6 expression is hepatocyte specific with increased Hnf6 localization to hepatocyte nuclei [8]. While the Stat5 protein and *Igf1* response is intact in GH-treated KO Sham and BDL liver, relative to PBS-treated Sham and BDL KO liver respectively, as expected, Hnf6 gene and protein (Fig 3C and 3D) expression is absent in KO liver. These data show that despite intact Stat5 pathways and *Igf1* expression in KO mice, the loss of Hnf6 impairs GH improvement of BDL apoptosis and fibrosis in KO liver, suggesting that GH-mediated protective response is mediated through Hnf6, independent of Igf1.

### 3. Hnf6 binds inhibitor of Apoptosis Protein gene *Ciap1* promoter

**Fig 4:**
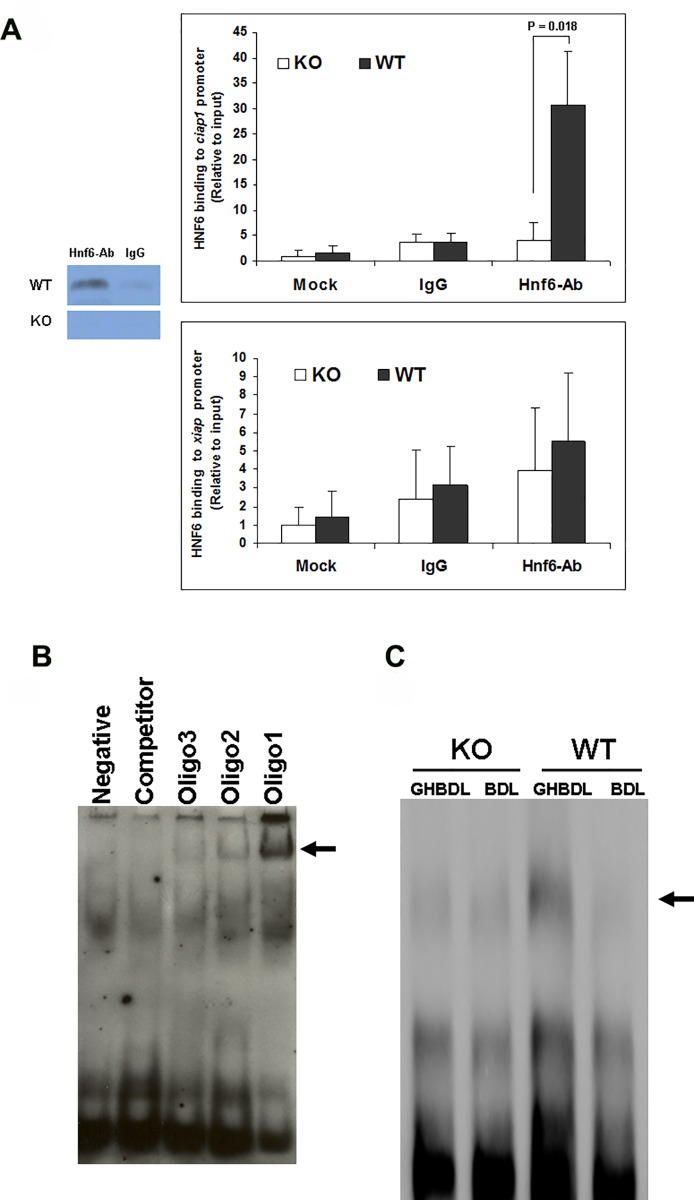
*Ciap1* promoter occupancy by Hnf6. (A) *Ciap1-* and *Xiap*-precipitated promoter fragments bound by mock, control IgG or anti-Hnf6 antibody were amplified by real time PCR and significant p value is shown. The gel insert shows precipitated anti-Hnf6 antibody treated Hnf6-DNA complex in WT vs KO nuclear extracts. Relative *Ciap1* promoter amplification is graphed relative to the corresponding WT mock data. (B) Electromobility shift assay EMSA of *in vitro* synthesized Hnf6 protein in the absence (Negative) or in the presence of 3 *Ciap1* promoter fragments with arrow showing the Oligonucleotide-Hnf6 protein precipitated complex. The Digoxin-labeled Oligonucleotide could be inhibited with DIG Oligo Competitor. (C) EMSA of nuclear extracts from KO and WT liver against *Ciap1* promoter oligonucleotide 1, with the arrow showing the location of the DNA-protein complex.

Genome-wide location analysis of Hnf6-bound genes by ChIP-on-ChIP assays of WT and KO liver (n = 3 each, S1 Table and S1 Fig) was performed to screen for potential Hnf6 target genes with antiapoptotic function. Hnf6 binding to *Ciap1* (also known as *Birc2*, Baculoviral Iap repeat-containing protein 2, a member of the Iap Inhibitor of Apoptosis Protein families involved in TNFa- and TRAIL (tumor necrosis factor-related apoptosis-inducing ligand)-induced cell signaling for apoptosis [25] [26] [27]) is markedly reduced in KO liver. To confirm the ChIP-on-ChIP results, we performed real-time PCR of WT and KO liver for apoptosis-related gene profiles. Among members of the Iap families, notably *Ciap1*, *Ciap2*, *Survivin*, and *Xiap* (Fig 3E), and *Bcl2* families (Fig 3F), only *Ciap1* and *Xiap* are significantly downregulated in KO liver. ChIP assays for constitutive *in vivo* binding of Hnf6 nuclear protein to *Ciap1* and *Xiap* promoters show that *Ciap1* promoter sequences are amplified from Hnf6-immunoprecipitated but not mock-treated or irrelevant IgG-treated hepatic chromatin/DNA complex in WT liver (Fig 4A). KO liver have no *Ciap1* promoter amplification, showing that baseline Hnf6 binding to *Ciap1* promoter in WT liver is lost in KO liver. No difference between IgG- vs Hnf6-bound *Xiap* complex is found in WT or KO liver. Furthermore, anti-Hnf6 antibody-precipitated *Xiap1* promoter amplification is similar to mock- or IgG-treated liver, consistent with the lack of constitutive binding of Hnf6 to *Xiap1* promoter.

*Ciap1* promoter sequence analysis for Hnf6 consensus binding motifs [28] reveals three potential HNF6 binding sites (Table 2). Electromobility shift assays show that *Ciap1* oligonucleotide 1 binding to *in vitro* synthesized Hnf6 protein is strongest (Fig 4B). These results further suggest that *Ciap1* is likely a direct Hnf6-regulated target gene.

**Table 2:** Mouse *Ciap1* promoter DNA sequences containing Hnf6 binding sites.

Name	Position	Sequence
Oligo 1	-539/-530	**ATATCgAcTT**
Oligo 2	-381/-372	**ATGTCCATTTG**
Oligo 3	+648/+657	**GTGTCAATT**c
**Hnf6 consensus sequence**		DHW**TC**Y**ATN**D

Lower case letters represent nucleotides deviating from Hnf6 consensus binding sequence. Nucleotide abbreviations are as follows: D is not C, H is A or T, W is A or G, Y is A or C and N is A, T, C or G.

### 4. *Ciap1* expression correlates with Hnf6 hepatic expression profile

**Fig 5:**
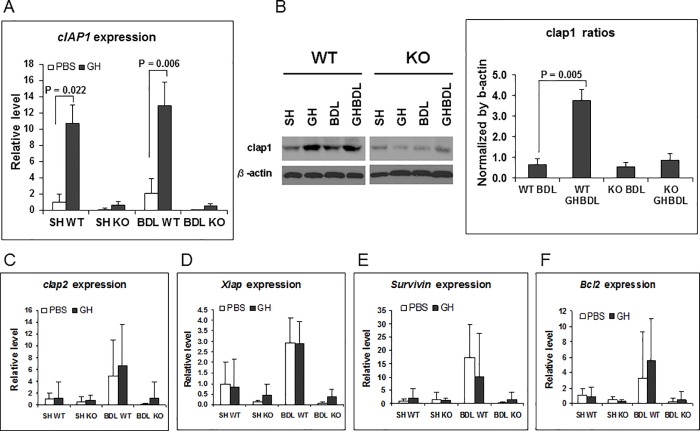
Iap gene and protein gene expression in GH-treated BDL liver. (A) Hepatic *Ciap1* expression in SH or BDL WT and BDL liver with PBS (□) or GH (■) treatment, with levels relative to SH WT liver and significant p values. (B) Representative western blot gel of SH or BDL WT and KO liver, and graph of quantitated signal from GH-treated WT and KO BDL liver with significant p value. (C-F) *Ciap2*, *Xiap*, *Survivin*, *Bcl2* expression in SH or BDL WT and KO liver with PBS (□) or GH (■) treatment, with levels relative to SH WT liver.

We next characterized the relationship between *in vivo* Hnf6 and hepatic expression of *Iap* during BDL. Previously, GH treatment reduces the extent of BDL-mediated downregulation of Hnf6 expression [8] (also Fig 3C and 3D). In the current study, GH treatment in Sham or BDL WT liver is associated with upregulation of hepatic *Ciap1* (Fig 5A) relative to PBS Sham or BDL liver respectively, but not of *Xiap*, *Survivin*, or *Ciap2* (Fig 5C–5E). In the KO liver, the loss of Hnf6 is associated with the loss of induction in the expression of all Iap family of genes during BDL, with or without GH treatment (Fig 5A). Of note, *Bcl2* gene expression in both WT and KO liver is similar between PBS-treated BDL and GH-treated BDL samples (Fig 5F). Western blotting shows that paralleling *Ciap1* expression pattern in WT liver, Ciap1 protein was higher in GH-treated Sham relative to PBS-Sham liver, and likewise, in GH-BDL liver relative to PBS-BDL liver (Fig 5B). In contrast, Ciap1 expression in KO liver is uniformly lower in Sham, PBS-BDL and GH-BDL relative to WT liver counterparts (Fig 5B). The data showing tight correlations between Hnf6 and Ciap1 patterns of expression are consistent with *Ciap1* as an Hnf6-regulated target gene.

### 5. Hnf6 binds to *Ciap1* promoter in BDL liver

Electromobility shift assay of liver nuclear extracts against *Ciap1* promoter oligonucleotide 1 (Fig 4C) shows that *in vivo*, Hnf6-*Ciap1* signal complex is stronger in GH-treated BDL WT liver relative to PBS-BDL WT liver, reflecting enhanced Hnf6 expression and Hnf6 binding to the *Ciap1* promoter in GH-treated samples. KO BDL liver shows poor HNF6-*Ciap1* signal complex, even with GH treatment. These data are in further support of Hnf6 binding and regulation of the *Ciap1* promoter as a target gene during the hepatic adaptive response to injury.

### 6. GH enhances Ciap1 and attenuates apoptosis in primary hepatocytes

**Fig 6:**
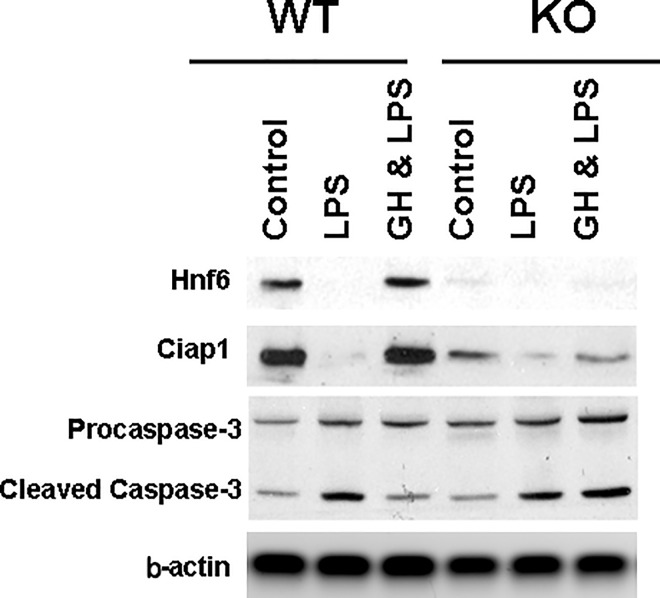
Apoptosis in LPS-treated WT and KO primary hepatocytes. Representative western blot of Hnf6, Ciap1, Procaspase-3, Cleaved Caspase-3, and b-Actin expression of total proteins from primary hepatocytes in WT and KO mice (n = 3–5) after treatment for 32 hours with 1 ug/ml of LPS, with or without GH cotreatment.

IAP proteins suppress TNFa– αnd TRAIL-induced cell death by inhibiting the activity of initiator and effector caspases [25] [26] [27]. Ciap1 knockdowns with either targeted SiRNA or antagonist to Ciap using endogenous or pharmacologic SMAC (Second Mitochondrial Activator of Caspases) increase stress-mediated apoptosis, while Ciap1 overexpression inhibits cell death in various cell-derived tissues [29] [30] [31] [32]. In primary rat hepatocytes, transfection with adenoviral vector expressing human CIAP1 suppresses LPS/cytokine mediated caspase-3 activation [33]. Conversely, hepatocyte cell lines as well as primary mouse hepatocytes treated either with ShRNA targeting Ciap1 or with SMAC mimetics become sensitized to apoptosis [34] [35]. To further evaluate Hnf6/*Ciap1* transcriptional link with hepatocyte-specific apoptosis, primary hepatocytes from KO and WT liver were exposed *in vitro* to high dose LPS. Western blot (Fig 6) shows, as expected, severe suppression of Hnf6 along with Ciap1 expression in KO hepatocytes. LPS treatment is associated with diminished Hnf6 expression in WT hepatocytes as an acute phase response (an observation of Hnf6 downregulation in acute injury during BDL [15], and in ethanol- or carbon tetrachloride-treated liver (data not shown)). GH treatment however restores Hnf6 expression in LPS hepatocytes, with Ciap1 expression pattern paralleling that of Hnf6 in LPS and GH-LPS samples. In WT hepatocytes, suppression of Hnf6/Ciap1 at high LPS dose is associated with enhanced cleaved caspase-3, while GH-dependent increased Hnf6/Ciap1 expression correlates with suppression of LPS-stimulated apoptosis. In spite of Hnf6 loss and parallel reduction in Ciap1 expression, KO hepatocytes respond to LPS with similar ability as in WT hepatocytes to induce caspase-3. In these KO hepatocytes however, GH effect on enhancing Hnf6 and Ciap1 is impaired. This is associated with compromised ability of GH to attenuate LPS-induced apoptosis. In summary, the stress response in KO mice is characterized by the loss of GH-mediated attenuation of apoptotic injury in LPS-treated primary hepatocytes because of suppressed Hnf6- and Ciap1 expression.

## Discussion

Hepatocyte apoptosis is a universal finding in all forms of liver injury, including cholestasis. We previously demonstrated that Hnf6 mediates hepatocyte survival functions such as hepatocyte cellular proliferation, hepatic glucose and cholesterol metabolism through Hnf6 transcriptional activation of its target genes [4–7,36]. We herein evaluated the role of Hnf6 in hepatocyte-specific apoptosis in the previous model of BDL by using mice with conditional inactivation of *Hnf6* in the liver. Null mice do not exhibit more severe hepatocyte apoptotic BDL injury, demonstrating that Hnf6 is not essential to protection against hepatic apoptosis. Hepatic *Igf1* during BDL injury is unimpaired in KO mice. KO mice thus may have compensated for Hnf6 loss through, as previously described, Igf-1 pro-survival effects on cell proliferation [37,38] and anti-apoptotic activity [39–41]. The loss of Hnf6 function in KO mice may be further counterbalanced by preserved expression of *Oc2* and *Oc3* paralogs, *Foxa2*, and notably by compensatory increases in *Hnf1b*. Hnf1b and Foxa2 have been demonstrated to have prosurvival [42] and antiapoptotic [43] function in hepatocytes, respectively. These transcription factors may cooperate with Igf1 to further alleviate KO liver susceptibility to hepatic cell death.

Prior cholestatic injury models in *Ghr*, *Ghr/Mdr2* or *Stat5/Mdr2* null mice have implicated GH-Stat5-Igf1 pathway in GH-mediated cell growth, survival, metabolism, and particularly protection against hepatocyte apoptosis [9] [10]. GH can also modulate hepatic gene function in an Igf1-independent manner through GH-Stat5 regulation of *Hnf6* promoter. We previously demonstrated that GH treatment during BDL in mice attenuates injury-related downregulation of Hnf6 expression. In these animals, enhanced Hnf6 expression is associated with improved hepatocyte proliferation, cholestasis, and cholesterol clearance. This protective response is linked to GH-Stat5 induction of *Hnf6* and Hnf6 *Cyclin D1* and *Cyp7a1* target genes [8]. Our current data show that with GH treatment, broad reduction in hepatic apoptosis (extrinsic and intrinsic pathways) and in cholestasis is observed in WT BDL mice. GH protective function is however abrogated in KO BDL mice, despite intact Stat5 and *Igf1* response. Hnf6 is therefore necessary for GH broad protection against apoptosis and cholestasis during BDL injury, independently of Igf1 [8].

A*lbumin*-Cre-mediated liver-specific inactivation of *Hnf6* affects both hepatocytes and cholangiocytes in our KO liver. While the contribution of Hnf6 inactivation in cholangiocytes cannot be excluded, the demonstrated GH-Hnf6 link to hepatic apoptosis is likely hepatocyte-dominant on the basis of hepatocyte-specific TUNEL immunostaining and primary hepatocyte data. Furthermore, hepatocytes constitute 85% of the liver cell mass while cholangiocytes only comprise 3–5% of the epithelial cell population. Interestingly, our previous work demonstrated that while Hnf6 has prosurvival effects on hepatocytes, Hnf6 impairs cholangiocyte proliferation [15]. While we do not observe baseline biliary duct abnormalities in the KO mice, it remains to be determined if the KO liver will respond during BDL injury to GH with an accentuated ductal proliferative reaction over biliary apoptosis.

The liver displays a sexual dimorphic response to GH [44], with Hnf6 participating in this sex-differential network to regulate many metabolic enzymes in a sex-specific fashion, such as *CYP2C12*, a member of cytochrome P450 family [45]. We have yet to determine if GH- and Hnf6-mediated responses to hepatocyte apoptosis are also sex-specific.

With respect to cholestasis, bile transport gene promoters contain Hnf6 binding sites (data not shown), suggesting that Hnf6-targeted genes may be involved in bile uptake and excretion. Further evaluation of Hnf6 regulation of cholestatic genes is pending. Overall, our data suggest that previously described aggravated hepatic injury in *Gh*-deficient mice is likely from dual Igf1 and Hnf6 defective functions. The implication is that preservation of both Igf1 and Hnf6 axes is important to hepatic survival responses against apoptosis and cholestasis. Future evaluations of cholestatic injury in double null *Igf1/Hnf6* against single *Igf1* or *Hnf6* null mice will shed further light on their relative biological importance.

Hepatocellular cell death is a driver and sensitizer for liver inflammation and fibrosis [46]. GH attenuation of apoptosis is therefore expected to improve BDL-fibrosis [8]. GH-dependent Igf1 is an important protective mechanism against fibrosis, since hepatic fibrosis is accelerated in Igf-deficient cholestatic mice [10], but attenuated following Igf1 replacement [21]. Despite a normal Igf1 response to GH during BDL, GH does not improve hepatic fibrosis in KO mice. The GH-Hnf6 regulated pathway, independently of Igf1, is therefore an alternative mechanism for GH protective effect in fibrosis. A potential mediator of this pathway is Hnf6-regulated *Tgfb2r* and *Ctgf* target genes. We previously demonstrated that *in vivo*, Hnf6-adenoviral vector suppresses *Tgfb2r* hepatic expression [8]. With GH treatment, increased Hnf6 and reduced BDL hepatic fibrosis are associated with diminished Tgfb2r gene and protein [8], suggesting that Hnf6 negatively regulates *Tgfb2r*. Furthermore, *Tgfb2r* signaling is increased in global Hnf6 null mice embryonic liver [47], while our ChIP-on ChIP data show over-amplified *Tgfb2r* promoter in KO relative to WT liver (data not shown). These data are consistent with Hnf6 as a transcriptional suppressor of *Tgfb2r* promoter function. Of note, pancreatic *Ctgf* expression was upregulated in mice with pancreas-specific Hnf6 inactivation [48], suggesting that HNF6 antifibrotic function may be mediated through its transcriptional inhibition of *Tgfb2r* and *Ctgf*. It remains to be seen if GH-dependent alleviation of fibrosis is secondary to suppression of apoptosis by GH/Igf1-, GH/Hnf6-dependent pathways, and/or through GH/Hnf6-regulated suppression of Hnf6 fibrogenic *Tgfb2r* or *Ctgf* target genes.

Among members of the IAP Inhibitor of Apoptosis Protein families modulating TNF-induced apoptosis and survival [25] [26] [27], *Ciap1* (*Bicr2*) promoter expression is markedly reduced in KO liver. Consistent with *Ciap1* as an Hnf6-transcriptionally regulated gene, ChiP assays reveal that Hnf6 constitutively binds *Ciap1* promoter while baseline hepatic Ciap1 expression is severely diminished in KO mice. Electromobility shift assays additionally show that GH treatment of WT BDL mice increases Hnf6-*Ciap1* nuclear complex signal over untreated WT BDL liver, with real time PCR and Hnf6 and Ciap1 protein expression recapitulating the gel shift data. All of these GH-enhancing effects are suppressed in Hnf6 KO mice. Together, the data are in support of Hnf6-regulated *Ciap1* transcriptional activation as an underlying mechanism for GH antiapoptotic function during BDL.

During injury, Ciap1 inhibits apoptosis signaling through a Caspase-activation and -recruitment domain, and several Baculo Iap repeat domains to interact with TNF-receptor associated factors TRAF2/3 and caspase-3, -7, -9 [25] [26]. Despite lower baseline Ciap1 expression in KO primary hepatocytes, LPS stimulates caspase expression equally well in both WT and KO hepatocytes, in support of the complexity of LPS-induced apoptosis. These *in vitro* findings are compatible with our *in vivo* data showing that BDL KO liver do not exhibit worse apoptotic injury. GH restores Ciap1 and attenuates apoptosis in WT but not KO primary hepatocytes, consistent with Ciap1 as a mediator of GH hepatic antiapoptotic function in a hepatocyte-specific fashion through Hnf6-dependent pathways. These data recapitulate our *in vivo* whole liver findings that the protective effect of GH on apoptosis is lost in Hnf6- and Ciap1-deficient KO BDL mice. It is worth noting that Ciap is implicated in viral clearance in mouse model of hepatitis B infection [49], as well as in chemoresistance [32,50,51], providing the clinical rationale for IAP antiviral [52] and antitumor therapies [53]. It remains to be determined if Ciap1 link to the protective effect of GH on *in vivo* hepatocyte apoptosis can be explored as a potential application for liver support therapies.

## Conclusion

Hnf6 is not necessary to protection against LPS-apoptotic injury in hepatocytes, or to hepatic protection against apoptosis in BDL cholestasis. Hnf6 is however is critical to GH prosurvival function during LPS-injury in primary hepatocytes, as well as in the cholestastic liver, to attenuate hepatocyte apoptosis independently of GH/Igf1 axis. This is likely mediated through Hnf6 transcriptional target gene *Ciap1*. The implication is that for patients with hepatic Igf1 dysfunction in clinical settings such as chronic liver diseases [54], therapeutic interventions using Hnf6 and/or Ciap1 gain-of-function approaches may diminish hepatocellular death, improve hepatocyte proliferation and enhance liver function.

## Supporting Information

S1 Fig Cluster analysis of Hnf6-bound genes. Cluster diagram of the Cluster Analysis showing Hnf6-bound genes. R^2^ measures Hierarchical cluster analysis using MAT (model-based analysis of tiling-array) scores with *Ciap1* (or *Birc2*, denoted with *) having the highest MAT-score (664), and significant variance.
(DOC)

S1 Table ChIP-on-chip Hnf6-bound genes by functional categories. The table depicts Hnf6-bound genes with Enrichment Scores ≥ 1.25 (corresponding to a ≤ 0.05) and Group Enrichment Score ranking by biological significance based on overall EASE (Expression Analysis Systematic Explorer) scores (corresponding to the one-tailed Fisher exact probability of gene over-representation by functional class). Baculoviral IAP repeat-containing 2 (NM_007465, *Birc2* or *Ciap1*) is shown in Group 4, with Enrichment Score of 1.59.
(DOC)

## Abbreviations

α-Sma: α-smooth muscle actin
BDL: Bile duct ligation
Bicr2: Baculoviral Iap repeat-containing protein 2
Ciap: Cellular inhibitor of apoptosis protein
Ctgf: Connective tissue growth factor
GH: Growth hormone
Hnf: Hepatocyte nuclear factor
IAP: inhibitor of apoptosis protein
Igf: Insulin-like growth factor
KO: Hnf6 conditional null mice
LPS: Lipopolysaccharide
Oc: Onecut
SH: Sham
Tgfb2r: Transforming growth factor b2 Receptor
Xiap: X-linked inhibitor of apoptosis protein

## References

[1] Woelfle J, Chia DJ, Rotwein P (2003) Mechanisms of growth hormone (GH) action. Identification of conserved Stat5 binding sites that mediate GH-induced insulin-like growth factor-I gene activation. J Biol Chem 278: 51261–51266. 10.1074/jbc.M309486200 14532269

[2] Lahuna O, Rastegar M, Maiter D, Thissen JP, Lemaigre FP, and Rousseau GG. (2000) Involvement of STAT5 (signal transducer and activator of transcription 5) and HNF-4 (hepatocyte nuclear factor 4) in the transcriptional control of the hnf6 gene by growth hormone. Mol Endocrinol 14: 285–294. 10.1210/mend.14.2.0423 10674400

[3] Clotman F, Lannoy VJ, Reber M, Cereghini S, Cassiman D, Jackemin P, et al (2002) The onecut transcription factor HNF6 is required for normal development of the biliary tract. Development 129: 1819–1828. 11934848 10.1242/dev.129.8.1819

[4] Costa RH, Kalinichenko VV, Holterman AX, Wang X (2003) Transcription factors in liver development, differentiation, and regeneration. Hepatology 38: 1331–1347. 10.1016/j.hep.2003.09.034 14647040

[5] Lannoy VJ, Decaux JF, Pierreux CE, Lemaigre FP, Rousseau GG (2002) Liver glucokinase gene expression is controlled by the onecut transcription factor hepatocyte nuclear factor-6. Diabetologia 45: 1136–1141. 10.1007/s00125-002-0856-z 12189444

[6] Wang M, Tan Y, Costa RH, Holterman AX (2004) In vivo regulation of murine CYP7A1 by HNF-6: a novel mechanism for diminished CYP7A1 expression in biliary obstruction. Hepatology 40: 600–608. 10.1002/hep.20349 15349898

[7] Tan Y, Yoshida Y, Hughes DE, Costa RH (2006) Increased expression of hepatocyte nuclear factor 6 stimulates hepatocyte proliferation during mouse liver regeneration. Gastroenterology 130: 1283–1300. 10.1053/j.gastro.2006.01.010 16618419 PMC1440887

[8] Wang M, Chen M, Zheng G, Dillard B, Tallarico M, Ortiz Z, et al (2008) Transcriptional activation by growth hormone of HNF-6-regulated hepatic genes, a potential mechanism for improved liver repair during biliary injury in mice. Am J Physiol Gastrointest Liver Physiol 295: G357–366. 10.1152/ajpgi.00581.2007 18511741 PMC2519853

[9] Stiedl P, McMahon R, Blaas L, Stanek V, Svinka J, Grabner B, et al (2015) Growth hormone resistance exacerbates cholestasis-induced murine liver fibrosis. Hepatology 61: 613–626. 10.1002/hep.27408 25179284 PMC4986903

[10] Blaas L, Kornfeld JW, Schramek D, Musteanu M, Zollner G, Gumhold J, et al (2010) Disruption of the growth hormone—signal transducer and activator of transcription 5—insulinlike growth factor 1 axis severely aggravates liver fibrosis in a mouse model of cholestasis. Hepatology 51: 1319–1326. 10.1002/hep.23469 20162728 PMC2976853

[11] Fickert P, Fuchsbichler A, Wagner M, Zollner G, Kaser A, Tilg H, et al (2004) Regurgitation of bile acids from leaky bile ducts causes sclerosing cholangitis in Mdr2 (Abcb4) knockout mice. Gastroenterology 127: 261–274. 15236191 10.1053/j.gastro.2004.04.009

[12] Zhang H, Ables ET, Pope CF, Washington MK, Hipkens S, Means AL, et al (2009) Multiple, temporal-specific roles for HNF6 in pancreatic endocrine and ductal differentiation. Mech Dev. 10.1016/j.mod.2009.09.006 PMC2783291 19766716

[13] Waxman DJ, Holloway MG (2009) Sex differences in the expression of hepatic drug metabolizing enzymes. Mol Pharmacol 76: 215–228. 10.1124/mol.109.056705 19483103 PMC2713118

[14] Lahuna O, Fernandez L, Karlsson H, Maiter D, Lemaigre FP, Rousseau GG, et al (1997) Expression of hepatocyte nuclear factor 6 in rat liver is sex-dependent and regulated by growth hormone. Proc Natl Acad Sci U S A 94: 12309–12313. 9356445 10.1073/pnas.94.23.12309 PMC24918

[15] Holterman AX, Tan Y, Kim W, Yoo KW, Costa RH (2002) Diminished hepatic expression of the HNF-6 transcription factor during bile duct obstruction. Hepatology 35: 1392–1399. 10.1053/jhep.2002.33680 12029624

[16] Rausa F, Samadani U, Ye H, Lim L, Fletcher CF, Jenkins NA, et al (1997) The cut-homeodomain transcriptional activator HNF-6 is coexpressed with its target gene HNF-3 beta in the developing murine liver and pancreas. Dev Biol 192: 228–246. 10.1006/dbio.1997.8744 9441664

[17] Glaser S, Wang M, Ueno Y, Venter J, Wang K, Chen H, et al (2010) Differential transcriptional characteristics of small and large biliary epithelial cells derived from small and large bile ducts. Am J Physiol Gastrointest Liver Physiol 299: G769–777. 10.1152/ajpgi.00237.2010 20576918 PMC2950684

[18] Castilla-Cortazar I, Garcia M, Muguerza B, Quiroga J, Perez R, Santidrian S, et al (1997) Hepatoprotective effects of insulin-like growth factor I in rats with carbon tetrachloride-induced cirrhosis. Gastroenterology 113: 1682–1691. 9352873 10.1053/gast.1997.v113.pm9352873

[19] Held MA, Cosme-Blanco W, Difedele LM, Bonkowski EL, Menon RK and Denson LA. (2005) Alterations in growth hormone receptor abundance regulate growth hormone signaling in murine obstructive cholestasis. Am J Physiol Gastrointest Liver Physiol 288: G986–993. 10.1152/ajpgi.00287.2004 15604202

[20] Mair M, Zollner G, Schneller D, Musteanu M, Fickert P, Gumhold J, et al (2010) Signal transducer and activator of transcription 3 protects from liver injury and fibrosis in a mouse model of sclerosing cholangitis. Gastroenterology 138: 2499–2508. 10.1053/j.gastro.2010.02.049 20193684

[21] Mirpuri E, Garcia-Trevijano ER, Castilla-Cortazar I, Berasain C, Quiroga J, Rodrizuez-Ortigoza C, et al (2002) Altered liver gene expression in CCl4-cirrhotic rats is partially normalized by insulin-like growth factor-I. Int J Biochem Cell Biol 34: 242–252. 11849991 10.1016/s1357-2725(01)00123-6

[22] Vanderpool C, Sparks EE, Huppert KA, Gannon M and Means AL. (2012) Genetic interactions between hepatocyte nuclear factor-6 and Notch signaling regulate mouse intrahepatic bile duct development in vivo. Hepatology 55: 233–243. 10.1002/hep.24631 21898486 PMC3235248

[23] Margagliotti S, Clotman F, Pierreux CE, Beaudry JB, Jacquemin P, Rousseau GG, et al (2007) The Onecut transcription factors HNF-6/OC-1 and OC-2 regulate early liver expansion by controlling hepatoblast migration. Dev Biol 311: 579–589. 10.1016/j.ydbio.2007.09.013 17936262

[24] Jacquemin P, Lannoy VJ, Rousseau GG, Lemaigre FP (1999) OC-2, a novel mammalian member of the ONECUT class of homeodomain transcription factors whose function in liver partially overlaps with that of hepatocyte nuclear factor-6. J Biol Chem 274: 2665–2671. 9915796 10.1074/jbc.274.5.2665

[25] Silke J, Vucic D (2014) IAP family of cell death and signaling regulators. Methods Enzymol 545: 35–65. 10.1016/B978-0-12-801430-1.00002-0 25065885

[26] Budhidarmo R, Day CL (2015) IAPs: Modular regulators of cell signalling. Semin Cell Dev Biol 39: 80–90. 10.1016/j.semcdb.2014.12.002 25542341

[27] Lemke J, von Karstedt S, Zinngrebe J, Walczak H (2014) Getting TRAIL back on track for cancer therapy. Cell Death Differ 21: 1350–1364. 10.1038/cdd.2014.81 24948009 PMC4131183

[28] Jacquemin P, Durviaux SM, Jensen J, Godfraind C, Gradwohl G, Guillemot F, et al (2000) Transcription factor hepatocyte nuclear factor 6 regulates pancreatic endocrine cell differentiation and controls expression of the proendocrine gene ngn3. Mol Cell Biol 20: 4445–4454. 10825208 10.1128/mcb.20.12.4445-4454.2000 PMC85812

[29] Qi Y, Xia P (2012) Cellular inhibitor of apoptosis protein-1 (cIAP1) plays a critical role in beta-cell survival under endoplasmic reticulum stress: promoting ubiquitination and degradation of C/EBP homologous protein (CHOP). J Biol Chem 287: 32236–32245. 10.1074/jbc.M112.362160 22815481 PMC3442554

[30] Vince JE, Wong WW, Khan N, Feltham R, Chau D, Ahmed AU, et al (2007) IAP antagonists target cIAP1 to induce TNFalpha-dependent apoptosis. Cell 131: 682–693. 10.1016/j.cell.2007.10.037 18022363

[31] Zhao D, Sun Y, Wei X, Liang H, Zhao L, Dong X, et al (2015) cIAP1 attenuates shear stress-induced hBMSC apoptosis for tissue-engineered blood vessels through the inhibition of the mitochondrial apoptosis pathway. Life Sci 137: 81–88. 10.1016/j.lfs.2015.07.011 26188594

[32] Jung SA, Park YM, Hong SW, Moon JH, Shin JS, Lee HR, et al (2015) Cellular inhibitor of apoptosis protein 1 (cIAP1) stability contributes to YM155 resistance in human gastric cancer cells. J Biol Chem 290: 9974–9985. 10.1074/jbc.M114.600874 25635055 PMC4400372

[33] Schoemaker MH, Ros JE, Homan M, Trautwein C, Liston P, Poelstra K, et al (2002) Cytokine regulation of pro- and anti-apoptotic genes in rat hepatocytes: NF-kappaB-regulated inhibitor of apoptosis protein 2 (cIAP2) prevents apoptosis. J Hepatol 36: 742–750. 12044523 10.1016/s0168-8278(02)00063-6

[34] Akazawa Y, Guicciardi ME, Cazanave SC, Bronk SF, Werneburg NW, Kakisaka K, et al (2013) Degradation of cIAPs contributes to hepatocyte lipoapoptosis. Am J Physiol Gastrointest Liver Physiol 305: G611–619. 10.1152/ajpgi.00111.2013 24008361 PMC3840239

[35] Guicciardi ME, Werneburg NW, Bronk SF, Franke A, Yagita H, Thomas G, et al (2014) Cellular inhibitor of apoptosis (cIAP)-mediated ubiquitination of phosphofurin acidic cluster sorting protein 2 (PACS-2) negatively regulates tumor necrosis factor-related apoptosis-inducing ligand (TRAIL) cytotoxicity. PLoS One 9: e92124 10.1371/journal.pone.0092124 24633224 PMC3954888

[36] Tan Y, Adami G, Costa RH (2002) Maintaining HNF6 expression prevents AdHNF3beta-mediated decrease in hepatic levels of Glut-2 and glycogen. Hepatology 35: 790–798. 10.1053/jhep.2002.32482 11915024

[37] Baserga R, Hongo A, Rubini M, Prisco M, Valentinis B (1997) The IGF-I receptor in cell growth, transformation and apoptosis. Biochim Biophys Acta 1332: F105–126. 9196021 10.1016/s0304-419x(97)00007-3

[38] Gatto M, Drudi-Metalli V, Torrice A, Alpini G, Cantafora A, Biotta I, et al (2008) Insulin-like growth factor-1 isoforms in rat hepatocytes and cholangiocytes and their involvement in protection against cholestatic injury. Lab Invest 88: 986–994. 10.1038/labinvest.2008.63 18607346 PMC2569860

[39] Pi-Chieh Wang K, Lee LM, Lin TJ, Sheen-Chen SM, Lin JW, Chiu WT, et al (2011) Gene transfer of IGF1 attenuates hepatocellular apoptosis after bile duct ligation. J Surg Res 167: 237–244. 10.1016/j.jss.2009.07.051 19926099

[40] Drudi Metalli V, Mancino MG, Mancino A, Torrice A, Gatto M, et al (2007) Bile salts regulate proliferation and apoptosis of liver cells by modulating the IGF1 system. Dig Liver Dis 39: 654–662. 10.1016/j.dld.2007.03.008 17531559

[41] Onori P, Alvaro D, Floreani AR, Mancino MG, Franchitto A, Guido M, et al (2007) Activation of the IGF1 system characterizes cholangiocyte survival during progression of primary biliary cirrhosis. J Histochem Cytochem 55: 327–334. 10.1369/jhc.6R7125.2006 17164408

[42] De Vas MG, Kopp JL, Heliot C, Sander M, Cereghini S, Haumaitre C. (2015) Hnf1b controls pancreas morphogenesis and the generation of Ngn3+ endocrine progenitors. Development 142: 871–882. 10.1242/dev.110759 25715395 PMC4352981

[43] Wang K, Brems JJ, Gamelli RL, Holterman AX (2013) Foxa2 may modulate hepatic apoptosis through the cIAP1 pathway. Cell Signal 25: 867–874. 10.1016/j.cellsig.2012.12.012 23275033

[44] Zhang Y, Laz EV, Waxman DJ (2012) Dynamic, sex-differential STAT5 and BCL6 binding to sex-biased, growth hormone-regulated genes in adult mouse liver. Mol Cell Biol 32: 880–896. 10.1128/MCB.06312-11 22158971 PMC3272977

[45] Delesque-Touchard N, Park SH, Waxman DJ (2000) Synergistic action of hepatocyte nuclear factors 3 and 6 on CYP2C12 gene expression and suppression by growth hormone-activated STAT5b. Proposed model for female specific expression of CYP2C12 in adult rat liver. J Biol Chem 275: 34173–34182. 10.1074/jbc.M004027200 10931833

[46] Koyama Y, Brenner DA (2015) New therapies for hepatic fibrosis. Clin Res Hepatol Gastroenterol 39 Suppl 1: S75–79. 26206573 10.1016/j.clinre.2015.06.011 PMC4734896

[47] Clotman F, Jacquemin P, Plumb-Rudewiez N, Pierreux CE, Van der Smissen P, Dietz HC, et al (2005) Control of liver cell fate decision by a gradient of TGF beta signaling modulated by Onecut transcription factors. Genes Dev 19: 1849–1854. 10.1101/gad.340305 16103213 PMC1186184

[48] Zhang H, Ables ET, Pope CF, Washington MK, Hipkens S, Means AL, et al (2009) Multiple, temporal-specific roles for HNF6 in pancreatic endocrine and ductal differentiation. Mech Dev 126: 958–973. 10.1016/j.mod.2009.09.006 19766716 PMC2783291

[49] Ebert G, Preston S, Allison C, Cooney J, Toe JG, Stutz MD, et al (2015) Cellular inhibitor of apoptosis proteins prevent clearance of hepatitis B virus. Proc Natl Acad Sci U S A 112: 5797–5802. 10.1073/pnas.1502390112 25902529 PMC4426461

[50] Arnt CR, Chiorean MV, Heldebrant MP, Gores GJ, Kaufmann SH (2002) Synthetic Smac/DIABLO peptides enhance the effects of chemotherapeutic agents by binding XIAP and cIAP1 in situ. J Biol Chem 277: 44236–44243. 10.1074/jbc.M207578200 12218061

[51] Bockbrader KM, Tan M, Sun Y (2005) A small molecule Smac-mimic compound induces apoptosis and sensitizes TRAIL- and etoposide-induced apoptosis in breast cancer cells. Oncogene 24: 7381–7388. 10.1038/sj.onc.1208888 16044155

[52] Lucifora J, Trepo C (2015) Hepatitis: After HCV cure, HBV cure? Nat Rev Gastroenterol Hepatol 12: 376–378. 10.1038/nrgastro.2015.103 26100368

[53] Saleem M, Qadir MI, Perveen N, Ahmad B, Saleem U, Irshad T, et al (2013) Inhibitors of apoptotic proteins: new targets for anticancer therapy. Chem Biol Drug Des 82: 243–251. 10.1111/cbdd.12176 23790005

[54] Bonefeld K, Moller S (2011) Insulin-like growth factor-I and the liver. Liver Int 31: 911–919. 10.1111/j.1478-3231.2010.02428.x 21733081

