# Supplementary material for: Growth Hormone Mediates Its Protective Effect in Hepatic Apoptosis through Hnf6
Source: PLoS One. 2016 Dec 9;11(12):e0167085. doi: 10.1371/journal.pone.0167085 (PMC5147851; doi:10.1371/journal.pone.0167085)

**Supporting information**

**S1. Figure 1.** **Cluster analysis of Hnf6-bound genes**

Cluster diagram of the Cluster Analysis showing Hnf6-bound genes. R^2^ measures Hierarchical cluster analysis using MAT (model-based analysis of tiling-array) scores with *Ciap1* (or *Birc2*, denoted with *) having the highest MAT-score (664), and significant variance compared


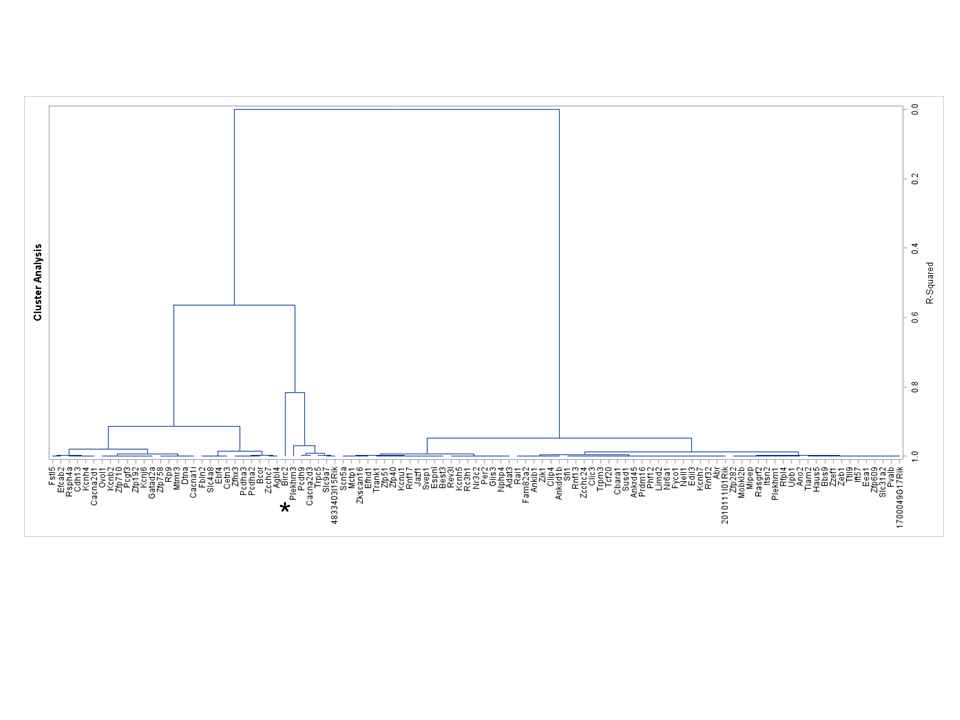

Supplement: S1 Fig — Cluster diagram of the Cluster Analysis showing Hnf6-bound genes. R2 measures Hierarchical cluster analysis using MAT (model-based analysis of tiling-array) scores with Ciap1 (or Birc2, denoted with *) having the highest MAT-score (664), and significant variance. (DOC) [file pone.0167085.s001.doc]
