# Supplementary material for: Growth Hormone Mediates Its Protective Effect in Hepatic Apoptosis through Hnf6
Source: PLoS One. 2016 Dec 9;11(12):e0167085. doi: 10.1371/journal.pone.0167085 (PMC5147851; doi:10.1371/journal.pone.0167085)
Supplement: S1 Table — The table depicts Hnf6-bound genes with Enrichment Scores ≥ 1.25 (corresponding to a ≤ 0.05) and Group Enrichment Score ranking by biological significance based on overall EASE (Expression Analysis Systematic Explorer) scores (corresponding to the one-tailed Fisher exact probability of gene over-representation by functional class). Baculoviral IAP repeat-containing 2 (NM_007465, Birc2 or Ciap1) is shown in Group 4, with Enrichment Score of 1.59. (DOC) [file pone.0167085.s002.doc]

**Supporting information**

**S1. Table.** **ChIP-on-chip** **Hnf6-bound genes by functional categories**

| **Group** | | **Enrichment Score** | | **Genebank ID** | | **Functional Annotation** | |  |
| --- | --- | --- | --- | --- | --- | --- | --- | --- |
| Group 1 | | Enrichment Score: 1.78 | | NM_001122998  NM_198895  NM_009027  NM_011365 | | T-cell lymphoma invasion and metastasis 2 active BCR-related gene  RAS protein-specific guanine nucleotide-releasing factor 2 intersectin 2 | |  |
| Group 2 | | Enrichment Score: 1.65 | | NM_178673  NM_026626  NM_013645  NM_198117  NM_028889  NM_007684  NM_153589  NM_144822  NM_019707  NM_001081377  NM_138662  NM_030174 | | follistatin-like 5  EF-hand calcium binding domain 2 parvalbumin  similar to protocadherins  EF hand domain containing 1 centrin 3  anoctamin 2  calcium binding atopy-related autoantigen 1 cadherin 13  protocadherin 9 protocadherin alpha 3  multiple C2 domains, transmembrane 1 | |  |
| Group 3 | | Enrichment Score: 1.6 | | NM_178415  NM_030207  NM_029064  NM_028680  NM_153424  NM_001033136  NM_027999  NM_001162957 | | Bardet-Biedl syndrome 9 (human)  Sfi1 homolog, spindle assembly associated (yeast) tubulin tyrosine ligase-like family, member 9 intraflagellar transport 57 homolog (Chlamydomonas) nephronophthisis 4 (juvenile) homolog (human)  family with sequence similarity 82, member A2  vomeronasal 2, receptor 53; predicted gene 8146; gene model 398, (NCBI)  radial spoke head 4 homolog A (Chlamydomonas) | |  |
| Group 4 | | Enrichment Score: 1.59 | | NM_030231  NM_172536  NM_010264  NM_172716  NM_011264  NM_139141  NM_001024952  NM_028538  NM_009558  NM_001001932  NM_007496  NM_145484  NM_009555  NM_009577  NM_028079  NM_175459  NM_178061  NM_001033043  NM_027504  NM_172397  NM_029008  NM_001045536  NM_001110513 | | ATP/GTP binding protein-like 4 zinc finger protein 609  nuclear receptor subfamily 6, group A, member 1 polycomb group ring finger 3  REV3-like, catalytic subunit of DNA polymerase zeta RAD54 like (S. cerevisiae)  zinc finger protein 192  RING CCCH (C3H) domains 1  RIKEN cDNA 1700049G17 gene  hypothetical protein LOC100044272; zinc finger protein 51 early endosome antigen 1  zinc finger homeobox 3  hypothetical protein LOC100044280; zinc finger protein 758 zinc finger protein 40  zinc finger protein interacting with K protein 1  RIKEN cDNA 2010111I01 gene  GLIS family zinc finger 3  MOB1, Mps One Binder kinase activator-like 2B (yeast)  ring finger protein 17  PR domain containing 16  similar to epithelial protein lost in neoplasm; LIM domain containing 2  RIKEN cDNA 4833403I15 gene  zinc finger, ZZ-type with EF hand domain 1 early B-cell factor 4 | |  |
|  | | | NM_001101433  NM_007465  NM_029510  NM_001145013  NM_001039493  NM_173406  NM_145596  NM_001099323  NM_027436  NM_138590  NM_183034  NM_207650  NM_146175  NM_001114140  NM_001083906  NM_011546  NM_001100606  NM_175433  NM_021470  NM_019935  NM_174852  NM_001003909  NM_148925  NM_028860  NM_009021  NM_011883  NM_133995  NM_018739  NM_011066 | | zinc finger, CCHC domain containing 24 baculoviral IAP repeat-containing 2  BCL6 interacting corepressor ret finger protein-like 4  pleckstrin homology domain containing, family M, member 3  JAZF zinc finger 1  similar to Transcriptional repressor p66 alpha; GATA zinc finger domain containing 2A  zinc finger with KRAB and SCAN domains 16  mitochondrial intermediate peptidase; similar to Mipep protein zinc finger, CCHC domain containing 7  pleckstrin homology domain containing, family M member 1 dystrobrevin alpha  zinc finger protein 282 transcription factor 20  nuclear receptor subfamily 3, group C, member 2 zinc finger E-box binding homeobox 1  adenosine deaminase, tRNA-specific 3, TAD2 homolog (S. cerevisiae)  zinc finger protein 710 ring finger protein 32  OVO homolog-like 1 (Drosophila) PHD finger protein 12  ankyrin repeat and IBR domain containing 1  FYVE and coiled-coil domain containing 1  predicted gene 5043; myotubularin related protein 3 retinoic acid induced 1  ring finger protein 13 ureidopropionase, beta  retinitis pigmentosa 9 (human)  period homolog 2 (Drosophila) | | | |
| Group 5 | | Enrichment Score: 1.57 | NM_028664  NM_001033292  NM_001164659  NM_030179  NM_026814  NM_001042714 | | ankyrin repeat domain 45 espin-like  lupus brain antigen 1  CAP-GLY domain containing linker protein family, member 4 dysferlin interacting protein 1  ankyrin repeat and death domain containing 1B | | | |
| Group 6 | | Enrichment Score: 1.4 | NM_025286  NM_027085  NM_001007583  NM_153589  NM_177353  NM_008432  NM_009428  NM_001025585  NM_172805  NM_001044308  NM_009784  NM_001081194  NM_021544  NM_021530  NM_001035243 | | solute carrier family 31, member 2 chloride intracellular channel 3 bestrophin 3  anoctamin 2  solute carrier family 9 (sodium/hydrogen exchanger), member 7 potassium channel, subfamily U, member 1  transient receptor potential cation channel, subfamily C, member 5 potassium inwardly-rectifying channel, subfamily J, member 6 potassium voltage-gated channel, subfamily H (eag-related), member 5 calcium channel, voltage-dependent, alpha 1I subunit  calcium channel, voltage-dependent, alpha2/delta subunit 1  potassium voltage-gated channel, subfamily H (eag-related), member 4 sodium channel, voltage-gated, type V, alpha  solute carrier family 4 (anion exchanger), member 8  transient receptor potential cation channel, subfamily M, member 3 | | | |
|  | | | NM_009785  NM_001098528  NM_133207 | | calcium channel, voltage-dependent, alpha2/delta subunit 3 potassium voltage gated channel, Shab-related subfamily, member 2 potassium voltage-gated channel, subfamily H (eag-related), member 7 | |  | |
| Group 7 | Enrichment Score: 1.3 | | NM_010103 | | EGF-like repeats and discoidin I-like domains 3 | |  | |
|  |  | | NM_022814 | | sushi, von Willebrand factor type A, EGF and pentraxin domain containing 1 | |  | |
|  |  | | NM_001037906 | | NEL-like 1 (chicken) | |  | |
|  |  | | NM_001163288 | | predicted gene 12528 | |  | |
|  |  | | NM_001081437 | | fibulin 2 | |  | |
| Group 8 | Enrichment Score: 1.25 | | NM_011864 | | 3'-phosphoadenosine 5'-phosphosulfate synthase 2 | |  | |
|  |  | | NM_153587 | | ribosomal protein S6 kinase, polypeptide 5 | |  | |
|  |  | | NM_011958 | | origin recognition complex, subunit 4-like (S. cerevisiae) | |  | |
|  |  | | NM_173185 | | casein kinase 1, gamma 1 | |  | |
|  |  | | NM_001031621 | | ATP-binding cassette, sub-family A (ABC1), member 17 | |  | |
|  |  | | NM_001033285 | | CDC42 binding protein kinase alpha | |  | |
|  |  | | NM_030091 | | Obg-like ATPase 1 | |  | |
|  |  | | NM_011160 | | protein kinase, cGMP-dependent, type I | |  | |
|  |  | | NM_009535 | | Yamaguchi sarcoma viral (v-yes) oncogene homolog 1 | |  | |
|  |  | | NM_134079 | | adenosine kinase | |  | |
|  |  | | NM_026458 | | ATP-binding cassette, sub-family A (ABC1), member 14 | |  | |
|  |  | | NM_010690 | | large tumor suppressor | |  | |
|  |  | | NM_011840 | | mitogen-activated protein kinase kinase 5 | |  | |
|  |  | | NM_144896 | | PET112-like (yeast) | |  | |
|  |  | | NM_007439 | | anaplastic lymphoma kinase | |  | |
|  |  | | NM_172734 | | predicted gene 3555; predicted gene 7451; serine/threonine kinase 38 like | |  | |
|  |  | | NM_007938 | | Eph receptor A6 | |  | |
|  |  | | NM_019497 | | G protein-coupled receptor kinase 4 | |  | |
|  |  | | NM_001160386 | | dynein, axonemal, heavy chain 7B; dynein, axonemal, heavy chain 7A | |  | |
|  |  | | NM_001166654 | | cyclin-dependent kinase-like 3 | |  | |
|  |  | | NM_029653 | | death associated protein kinase 1 | |  | |
|  |  | | NM_009014 | | RAD51-like 1 (S. cerevisiae) | |  | |
|  |  | | NM_008846 | | phosphatidylinositol-4-phosphate 5-kinase, type 1 beta | |  | |
|  |  | | NM_013764 | | deoxyguanosine kinase | |  | |
|  |  | | NM_027498 | | similar to Serine/threonine-protein kinase QSK; cDNA sequence BC033915 | |  | |
|  |  | | NM_175031 | | serine/threonine kinase 36 (fused homolog, Drosophila) | |  | |
|  |  | | NM_001166030 | | myosin light chain kinase family, member 4 | |  | |
|  |  | | NM_021462 | | MAP kinase-interacting serine/threonine kinase 2 | |  | |
|  |  | | NM_029031 | | sedoheptulokinase | |  | |
|  |  | | NM_001161665 | | kinesin family member 26B | |  | |
|  |  | | NM_011948 | | mitogen-activated protein kinase kinase kinase 4 | |  | |
|  |  | | NM_027539 | | doublecortin-like kinase 2 | |  | |
|  |  | | NM_015755 | | similar to putative serine/threonine protein kinase MAK-V | |  | |
|  |  | | NM_001081357 | | mitogen-activated protein kinase kinase kinase kinase 3 | |  | |
|  |  | | NM_175538 | | RIKEN cDNA E130304F04 gene; hypothetical protein LOC100040601 | |  | |
|  |  | | NM_013785 | | inositol hexaphosphate kinase 1 | |  | |
|  |  | | NM_013845 | | receptor tyrosine kinase-like orphan receptor 1 | |  | |
|  |  | | NM_008855 | | protein kinase C, beta | |  | |
|  |  | | NM_010154 | | v-erb-a erythroblastic leukemia viral oncogene homolog 4 (avian) | |  | |
|  |  | | NM_031386  NM_015771 | | testis expressed gene 14  large tumor suppressor 2 | |  | |

The table depicts Hnf6-bound genes with Enrichment Scores ≥ 1.25 (corresponding to a < 0.05) and Group Enrichment Score ranking by biological significance based on overall EASE (Expression Analysis Systematic Explorer) scores (corresponding to the one-tailed Fisher exact probability of gene over-representation by functional class). Baculoviral IAP repeat-containing 2 (NM_007465, *Birc2* or *Ciap1*) is shown in Group 4, with Enrichment Score of 1.59.
